# Analgesic Effect of Butorphanol during Castration in Donkeys under Total Intravenous Anaesthesia

**DOI:** 10.3390/ani11082346

**Published:** 2021-08-09

**Authors:** Paola Straticò, Augusto Carluccio, Vincenzo Varasano, Giulia Guerri, Riccardo Suriano, Domenico Robbe, Ilaria Cerasoli, Lucio Petrizzi

**Affiliations:** 1 Faculty of Veterinary Medicine, University of Teramo, Località Piano D’Accio, 64100 Teramo, Italy; pstratico@unite.it (P.S.); acarluccio@unite.it (A.C.); vvarasano@unite.it (V.V.); suriano_riccardo@hotmail.it (R.S.); drobbe@unite.it (D.R.); lpetrizzi@unite.it (L.P.); 2 Clinica Veterinaria Borghesiana, Via di Vermicino 96, 00133 Roma, Italy; ilaria.cerasoli@gmail.com

**Keywords:** anaesthesia, analgesia, butorphanol, castration, detomidine, donkey

## Abstract

**Simple Summary:**

Despite the growing interest in donkey’s welfare, there is little scientific evidence in this species about the analgesic effect of the drugs most used in horses. Total intravenous anaesthesia is a valuable option for field surgery such as castration. Moreover, it is often preferred in small donkeys due to the anatomical features of their proximal airways. The aim of this study was to evaluate the effect of a low dose of butorphanol, in donkeys undergoing castration, after sedation with detomidine before total intravenous anaesthesia with a mixture of guaifenesin, ketamine and detomidine. The addition of butorphanol to detomidine produced more muscle relaxation, and reduced mean heart rate and blood pressure. This resulted in a reduced requirement for rescue analgesia, a more superficial anaesthetic plan and a shorter surgical time. It can be concluded that butorphanol allowed a more stable and superficial anaesthetic plan, probably due to its analgesic effect and synergistic action with detomidine. Due to the low dosages of drugs used and the absence of adverse effects, this protocol can be considered valuable in field conditions and for short surgical procedures, where safety equipment is not available.

**Abstract:**

Pain management is necessary for all surgical procedures. Little scientific evidence about drug efficacy in donkeys is available. The aim of this study was to evaluate the analgesic effect of butorphanol in donkeys undergoing orchiectomy under total intravenous anaesthesia with guaifenesin-ketamine-detomidine. A randomized blinded prospective clinical trial (Protocol n. 2021/0000338), was carried out on 18 clinically healthy donkeys undergoing bilateral orchiectomy. Patients were assigned to Group D (*n* = 8) or Group DB (*n* = 10) if receiving intravenous detomidine or detomidine-butorphanol respectively, before induction of general anaesthesia with ketamine-diazepam. Intraoperative muscle relaxation, nystagmus, palpebral reflex, heart and respiratory rate, and non-invasive blood pressure were evaluated every 2 min; time to prepare the patient, duration of surgery and anaesthesia and recovery score were recorded. Group D had significantly longer surgical time, higher heart rate, higher systolic and mean blood pressure (*p* < 0.05; repeated measure ANOVA), increased muscle rigidity and expression of palpebral reflex (*p* < 0.05; Mann–Whitney U test) than group DB. Top-ups with thiopental were statistically higher in Group D. Butorphanol and detomidine together produced a more stable anaesthetic plan. The low dosage of opioid and alpha-2-agonists and reduced rescue anaesthesia are responsible for a safer and more superficial anaesthesia, which is mandatory under field conditions.

## 1. Introduction

Despite the growing interest in equids other than the horse, there is little scientific evidence of the efficacy of anaesthetic drugs regarding donkeys (*Equus asinus*) and mules 41 [1,2,3]. Currently the importance of such animals is expanding not only in developing countries [4,5,6], but also in Europe as a result of animal-assisted therapies and interventions [7,8]. While it is known that the pharmacological profiles of analgesics in donkeys are clinically different from horses [9,10], very few drugs are registered for this species. Conversely, many drugs are used off-label, extrapolating doses from those determined for horses. However, the impact of interspecies differences in pharmacokinetics and pharmacodynamics of drugs is important and should be considered. Donkeys usually require higher dosages than horses. Indeed, they have a faster metabolism for most analgesics and anaesthetics, with a shorter dosing interval [1,2]. The only drug that does not follow this rule is guaiphenesin, which, although cleared faster in donkeys than in horses, requires lower doses to produce recumbency [1,11].

Most of the procedures required for the health management of donkeys (castration, foot disorders, dental treatments, tumor removal) require sedation and analgesia, with or without general anaesthesia [12]. Under field conditions, inhalation anaesthesia can be challenging due to the unavailability of anaesthetic equipment and the need for appropriate medical care in the case of complications, such as severe cardiovascular and respiratory depression. As a rule of thumb, in field settings, drugs with minimal side-effects, but with good analgesic and sedative effects are generally required [13] and if general anesthesia is necessary, total intravenous anaesthesia (TIVA) must be preferred.

Detomidine is a specific alpha_2_-adrenoreceptor agonist, although high doses also produce activation of alpha_1_ adrenoreceptors. Compared to xylazine and medetomidine, it is responsible for a longer and more profound sedation and analgesia in horses [14,15]. As for horses, detomidine has a dose-dependent sedative effect in donkeys, although higher doses are required [12,16,17].

Butorphanol is an agonist-antagonist opioid [18,19,20]. It is commonly used in combination with alpha_2_-adrenoreceptor drugs, mostly detomidine, for their excellent synergistic effect [20,21,22,23]. Its analgesic effect is dose-dependent with a mean duration in ponies of 15–90 min at 0.05–0.4 mg/kg bwt [24]. TIVA is considered a valuable option if inhalation anaesthesia is not available. The association of alpha_2_-agonists and muscle relaxants produces less cardiovascular depression in ponies and horses [25,26,27,28,29,30,31]. A variety of TIVA protocols have been set up for different kinds of surgery in horses, although little is documented for donkeys and mules [32,33]. This type of general anaesthesia is generally preferred in miniature or in young donkeys because of the difficult orotracheal intubation, related to the different anatomical features of the larynx compared to horses [34].

Donkeys are also usually presented for castration at an older age, with an increased size of the testes, larger vessels and an increased amount of scrotal and inguinal fat; these factors can facilitate post-operative complications particularly when a standing approach is used [35]. In addition, TIVA protocols in donkeys are preferred because of the reduced cost compared to inhalation anaesthesia, the availability of inhalation anaesthesia under field conditions (which is usually preferred by owners) and the cardiorespiratory safety [36].

The aim of this study was to evaluate the efficacy of butorphanol on the need to top-up drugs and on the clinical cardiovascular and respiratory response to the surgical stimulus in donkeys undergoing orchiectomy under TIVA with guaifenesin-ketamine-detomidine.

## 2. Materials and Methods

The study was approved by the O.p.B.A. of the Istituto Zooprofilattico of Teramo “G. Caporale” (Protocol n. 2021/0000338) and carried out on donkeys referred for bilateral orchiectomy to the Veterinary Teaching Hospital of the University of Teramo. The study design was a randomized blinded prospective clinical trial, where the anesthetist and personnel in charge of monitoring anaesthesia were unaware of the treatment. Inclusion criteria were a clinically healthy status (ASA 1 classification of patients) and the scrotal localization of both testes.

The donkeys were considered healthy based on clinical examination and results of a complete cell blood count and serum biochemical analysis. Baseline rectal temperature, heart rate (HR), and respiratory rate (RR) were recorded. Food, but not water, was withheld for 12 h before surgery.

After a 14-gauge 48 mm catheter was aseptically placed into the left jugular vein, donkeys were randomly administered intravenously (i.v.) detomidine (10 µg/kg, Domidine^®^; Dechra, Northwich, UK) (Group D) or detomidine-butorphanol (10 µg/kg + 50 µg/kg, Nargesic^®^; ACME Srl, Reggio Emilia, Italy) (Group DB) before induction of general anaesthesia with ketamine and diazepam (2.2 mg/kg+ 20 µg/kg, Ketavet 100^®^; ACME Srl, Reggio Emilia, Italy, Ziapam^®^; Dechra, Northwich, UK). In group DB, butorphanol was administered 5 min after the first bolus of detomidine, whereas donkeys of Group D received an equal volume of saline.

When an adequate state of sedation was obtained (clear lethargy, drop of the head and lack of response to external stimuli) [37,38], general anaesthesia was induced in a padded box and maintained with TIVA with a mixture of guaiphenesin (80 mg/mL, Knock Out^®^; ACME Srl, Reggio Emilia, Italy), ketamine (2 mg/mL) and detomidine (20 µg/mL) (GKD) at the Constant Infusion Rate (CRI) of 1 mL/kg/h administered through a volumetric infusion set (Infusomat fmS; B-Braun, Melsungen, Germany). To obtain a solution of GKD, 1 g of ketamine and 10 mg of detomidine were added to a 500-mL bag of 8% guaiphenesin. Once general anaesthesia was induced, the donkeys were placed in dorsal recumbency on a surgery table and moved into a surgery room, where the orchiectomies were performed. TIVA was initiated once proper positioning was achieved. Once positioned, the donkeys were prepared for surgery. Preparation of the patient (from correct positioning to the beginning of surgery) included trichotomy and surgical scrub and was performed in the surgery room.

Donkeys were maintained under spontaneous breathing, and oxygen supplementation was not provided.

The depth of anaesthesia was monitored every 2 min during surgery since TIVA was initiated, evaluating *muscle relaxation* (score 0–3; 0 = muscle relaxation present in the trunk and limbs; 1 = muscle twitching present in some regions of the trunk and limbs; 2 = muscle twitching present over the majority of the trunk and limbs; 3 = muscle rigidity present over the majority of the trunk and limbs) [39], *nystagmus* (0–2; 0 = no nystagmus, 1 = arrhythmic jerks, with high frequency, small amplitude, constant spatial planae and inconstant direction, 2 = rhythmic jerks, with constant amplitude, direction and spatial planae, decreasing frequency and constant duration) [40], *palpebral reflex* (0–2; 0 = no reflex, 1 = induced reflex, 2 = spontaneous reflex), HR (GE 850 Anaesthesia Monitor, GE Healthcare, Little Chalfont, UK), RR, and *non-invasive blood pressure* (NIBP) (GE 850 Anaesthesia Monitor, GE Healthcare, Little Chalfont, UK) through a cuff placed over the left brachial artery (Dura-Cuf^®^ 23–33 cm; Critikon Blood Pressure Cuffs, GE Healthcare, Little Chalfont, UK) [37]. Intraoperative RR was monitored by evaluating thoraco-lumbar expansion.

Once in dorsolateral recumbency, baseline values were collected for HR, RR, and NIBP. In the meantime, the surgical field was aseptically prepared, and afterwards a closed scrotal orchiectomy was performed. The right testis was firmly grasped, and an incision (Ts) was made along the median raphe of the scrotum. After exteriorization of the vaginal process, isolation of the spermatic cord and cremaster muscles from the connective tissue were provided through a blunt dissection of the scrotal ligament. After traction of the spermatic cord (Tt) and application of a polyglactin 910 2 USP transfixing suture (Vicryl^®^; Eticon Inc, Cornelia, USA) (Tl) as close as possible to the inguinal ring, a Serra emasculator was used to crush the cord (Te), which was transected after 3 min from the application of the emasculator. The timepoints Tt-Tl-Te were repeated first for the right and then for the left testicle. The surgical wound was left to heal by secondary intention.

A top-up anaeshesia plan was set up as follows: in case of poor muscle relaxation, an additional dose of thiopental (Pentothal sodium^®^; Intervet Italia Srl, Milano, Italy) (0.3 mg/kg i.v.) was administered. Concerning the rescue analgesia, when nystagmus or increased HR and/or MBP (mean arterial blood pressure) above 20% from the baseline were observed, an additional dose of ketamine (0.3 mg/kg i.v.) was preferred. The need for top-up anaesthesia with thiopental was set at a muscle relaxation score of 1–2. The need for rescue anaesthesia with ketamine was set at a nystagmus score of 1–2.

TIVA was interrupted when the Backhaus forceps were removed from the surgical field.

The number of additional doses of thiopental and/or ketamine was recorded and compared between group D and DB. Every time a top-up agent was administered, the surgeon was asked to stop the surgical stimulation until muscle relaxation was restored or nystagmus disappeared.

The donkeys were allowed to recover form anaesthesia without any assistance and the entire recovery phase was timed and scored from 1 to 3 (1 = Good: donkey stands after 1–3 attempts; no ataxia; 2 = Acceptable: more than 3 attempts to stand; mild, short-term ataxia; 3 = Poor: more than 3 attempts to stand; mild, substantial ataxia) [25].

The duration of anaesthesia (from induction to the end of TIVA), the time required to prepare the patient, the surgical time (from skin incision to removal of Backhaus forceps from the surgical field) and the time to recover from anaesthesia (from lateral recumbency in the recovery box to the standing position) were recorded for each donkey.

All donkeys received a broad-spectrum antibiotic therapy (penicillin-dihydrostreptomycin 10.000 U.I./kg i.m., every 12 h) (Repen^®^; FATRO S.p.A., Bologna, Italy) just before surgery and for the following 3 days. At the end of surgery, anti-inflammatory therapy (flunixin meglumine, 1 mg/kg i.m., every 12 h) (Flunifen^®^; Ceva Santé Animale, Libourne, France) was initiated, lasting for 3 days.

Normality was determined using the Shapiro–Wilk test. For the within-treatment comparisons of the parametric data, a repeated measure analysis of variance was used, followed by Bonferroni *post-hoc* test; non-parametric data were analyzed by Friedman test. Comparisons between Group D and Group DB were analyzed using a repeated measure ANOVA; non-normally distributed data were analyzed with Mann–Whitney U test. Parametric data are reported as mean ± standard deviation (SD), non-parametric data as median and range. The *p* value was set at 0.05.

Statistical analysis was performed using IBM SPSS v. 27. The confidence interval was set at 95%.

## 3. Results

Eighteen donkeys were included in the study, 8 were assigned to Group D and 10 to Group DB. No statistical differences in age and body weight were found between the groups (Group D: 25 ± 6 months; Group DB: 22 ± 10 months) (Group D: 158 ± 46 kg; Group DB: 157 ± 73 kg) (*p* = 0.385; *p* = 0.982) (Table 1).

**Figure 1:**
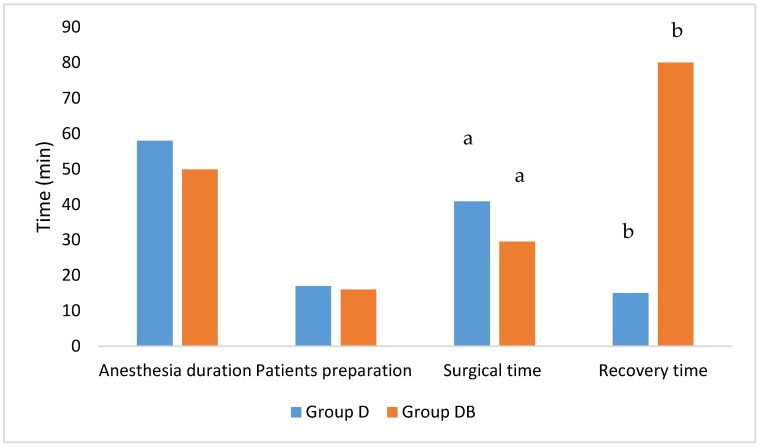
Vertical bar chart showing anaesthesia duration, patient preparation, surgical time and recovery time in the two groups. All the variables are expressed in function of time (minutes on the Y axis). Statistical differences are expressed by the same letter on the bars (a: *p* = 0.016; b: *p* = 0.003).

The mean duration of anaesthesia was not statistically different between groups, although it was longer for Group D (*p* = 0.232) (Table 1; Figure 1).

**Figure 2:**
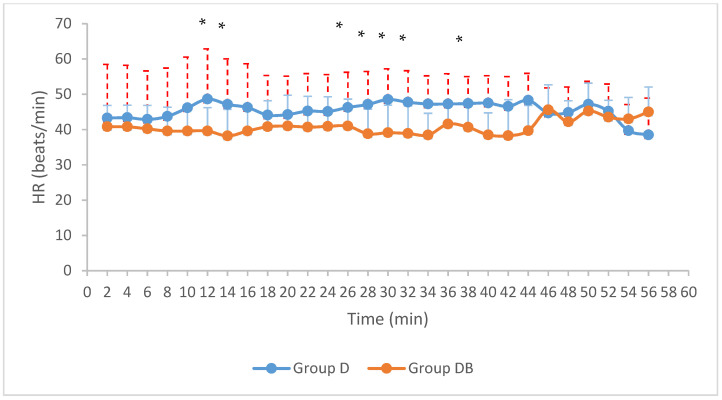
Mean HR for both groups. Values are expressed in beats per minute as a function of time (*: *p* < 0.05). SD is displayed (HR: heart rate; SD: Standard Deviation). Group D: group detomidine; Group DB: group detomidine-butorphanol.

On the other hand, surgical time was significantly longer in Group D than Group DB (*p* = 0.016), which also showed shorter time to recover completely from anaesthesia as compared to Group DB (*p* = 0.003) (Table 1; Figure 2).

No statistical differences were found when comparing the time required to prepare the patient (*p* = 0.723) (Table 1; Figure 1).

The mean HR was statistically higher in Group D for most of the duration of the procedure (T12,T14,T26,T28,T30,T32,T38) (Figure 2) (*p* = 0.011, *p* = 0.035, *p* = 0.049, *p* = 0.04, *p* = 0.014, *p* = 0.015, *p* = 0.022), whereas no significant differences in RR were found (Group D: 26 ± 3 breaths/min; Group DB: 23 ± 4 breaths/min) (0.210 < *p* < 0.935).

**Figure 3:**
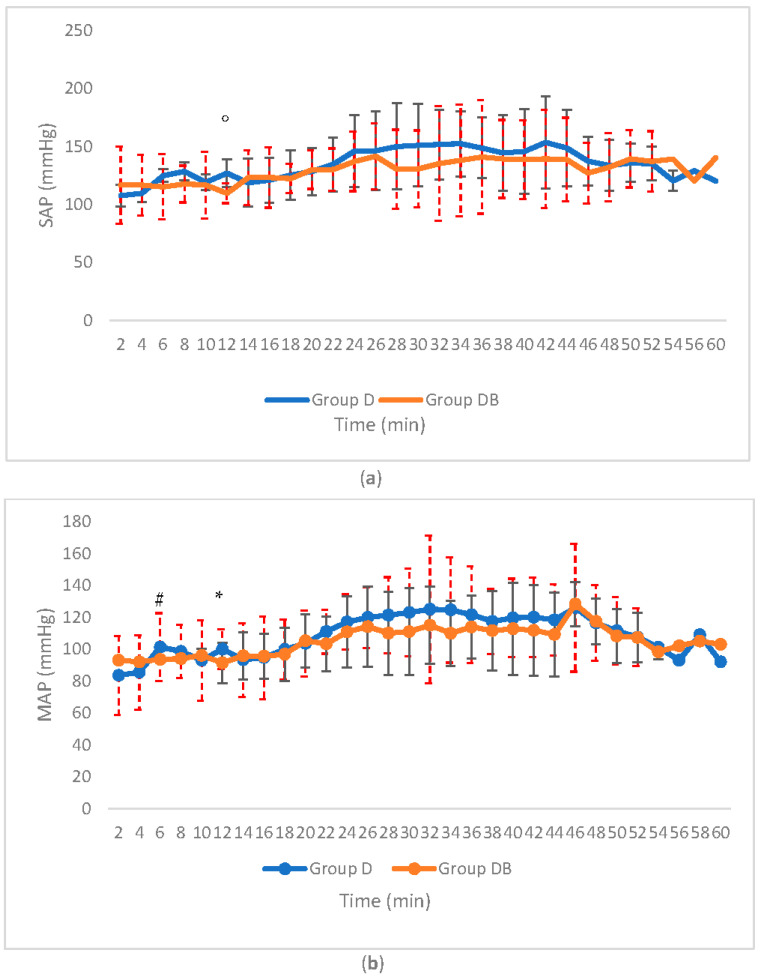
Line graph showing significant differences in SAP (**a**) and MAP (**b**) Statistical differences are expressed as same letter on the bars (°: *p* = 0.004; #: *p* = 0.022; *: *p* = 0.05). SD is displayed (SAP: Systolic Arterial Blood Pressure; MAP: Mean Arterial Blood Pressure; SD: Standard Deviation). Group D: group detomidine; Group DB: group detomidine-butorphanol.

When analyzing blood pressure, a significant difference was found in systolic and mean arterial blood pressure (SAP) and MAP, whereas no differences were present for diastolic arterial blood pressure (DAP, 0.242 < *p* < 0.971) (SAP at T12: Group D 127 mmHg vs. Group DB 110 mmHg, *p* = 0.04; MAP at T6,T12: Group D 110–120 mmHg vs. Group DB 93–94 mmHg, *p* = 0.022, *p* = 0.016) (Figure 3a,b).

The muscle relaxation score for Group D ranged from 0–2, whereas in Group DB it ranged from 0–1. The palpebral reflex score for Group D ranged from 0–3, whereas in Group DB it ranged from 0–2. In both cases the median was 0. Depth of anaesthesia was lower at T14 in Group D, showing muscle twitching and rigidity at that time point (*p* = 0.027). At the same time, Group D had a significantly higher expression of spontaneous palpebral reflex (*p* = 0.033).

**Figure 4:**
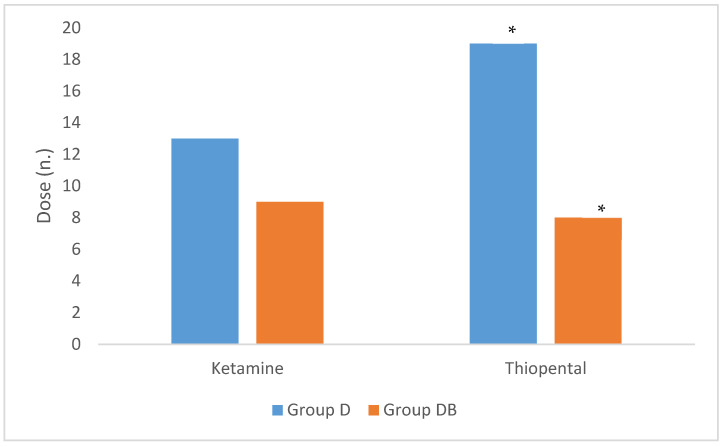
Total number of additional doses of ketamine and thiopental required to deepen anaesthesia in donkeys undergoing castration, randomized to receive an intravenous premedication with detomidine (Group D, n.8) or detomidine -butorphanol (Group DB, n.10). Asterisks (*) indicate the level of significance set at *p* < 0.05.

When considering the number of additional doses of ketamine and thiopental, we observed a higher requirement of thiopental in group D (19 in Group D, median 2, range 1–6; 8 in Group DB, median 0, range 0–5) (*p* = 0.012), whereas additional boluses of ketamine where not statistically different among groups (*p* = 0.116), although they were higher in Group D (13 in Group D, median 1.5, range 0–3; 9 in Group DB, median 0.5, range 0–4) (Figure 4) (*p* > 0.05). The percentage of donkeys of Group D that required at least 1 top-up of either ketamine or thiopental was 87.5% and 100%, respectively, whereas this percentage was 50% and 30% in Group DB for ketamine and thiopental, respectively.

The recovery score was good (score 1) in all donkeys.

The variables HR, RR, SBP, MBP, DBP, muscle relaxation, nystagmus and palpebral reflex were compared between groups at Ts, Tt, Tl Te for each testicle, but no statistical differences were found (*p* > 0.05). Looking at the time after the surgical stimulations, statistical differences were found 90–120 s after the stimulation itself.

**Figure 5:**
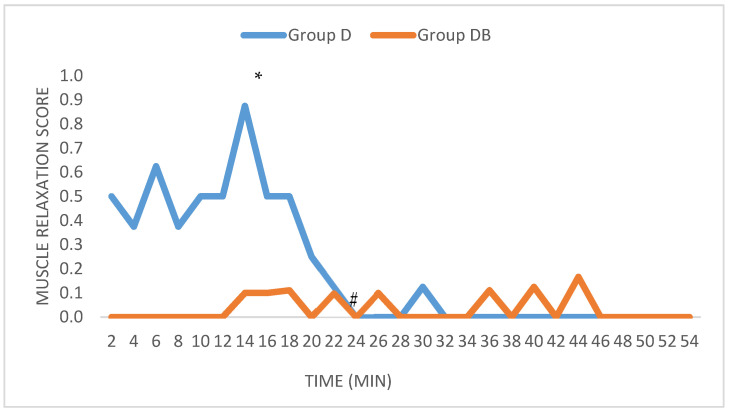
Trend of muscle relaxation during the surgical procedure in Group D (* is significantly higher than #; *p* = 0.03).

When each variable was analyzed for significative differences within each group, only muscle relaxation was significantly higher in Group D at T14 compared to T22-T28 (Figure 5) (*p* = 0.03).

## 4. Discussion

The present study aimed to demonstrate how a single dose of butorphanol during sedation with detomidine, provided better cardiovascular stability during bilateral orchiectomy in donkeys under a TIVA protocol including guaiphenesin-ketamine-detomidine. The association of a low dose of detomidine and butorphanol resulted in a more stable anaesthetic depth and lower number of additional doses of thiopental and ketamine, probably thanks to the analgesic effect of butorphanol.

Better understanding of pain management in donkeys is necessary and stands as an ethical requirement for practitioners [41,42,43]. Indeed, castration is demonstrated to be associated with a significant degree of pain that has a negative impact on patients’ well-being, compromising immune response and wound healing [44,45,46,47].

We decided to use detomidine rather than xylazine because its sedative and analgesic properties are of greater magnitude and longer duration [14,48]. Although only few reports about its effect on donkeys are available in the literature [13,16,37,49,50,51], detomidine is demonstrated to have a good sedative effect in horses, with a duration that is proportional to the dosage, with higher dosages (40 μg/kg) lasting up to 90 min [52]. The 10 μg/kg dosage of detomidine has similar sedative power, although the action is shorter (about 20 min) [13,14].

Butorphanol has also been widely studied in horses, with reports describing, as for detomidine, a dose-related analgesic effect, in terms of duration and depth [24,41,53,54,55,56,57,58,59,60,61]. The analgesic effects of low doses of detomidine in donkeys is still under debate. If a 10 μg/kg dosage of the drug provides a good sedative and analgesic effect in a horse [14,61], the same dose obtains a good sedative result in donkeys, but not enough analgesia for standing procedures when used alone [13,62]. Compared to medetomidine, which is not registered of use in equids in all countries, it has a longer lasting, although less potent behavioral effect [15].

The association of detomidine and butorphanol in equids is demonstrated to be a valuable and safe tool for pharmacological restraint, allowing one to reduce the adverse effects mediated by the use of a high amount of alpha-2 agonists [1,13,30,63,64,65]. In the literature, the dosage of detomidine and butorphanol ranges from 5–40 µg/kg and 25–50 µg/kg, respectively. Evaluation of their synergistic effect was usually achieved in the standing sedated animal, watching at the time of onset and depth of sedation, at clinicophysiological variables and nociception. In this study we meant to evaluate the association of reported dosages of detomidine and butorphanol in the recumbent and anaesthetized donkey undergoing surgical stimulation.

Despite the increasing knowledge about pain manifestation in donkeys and the development of new pain scales [66,67], we did not perform a pain score assessment before and after sedation, since this went beyond the aim of our study, instead focusing on intraoperative signs of pain. Pulse rate and mean blood pressure together with nystagmus, tearing, unstimulated closure of the eyelids, shivering, and tightening of muscles and electroencephalographic modifications are used as intraoperative markers of nociception and their variation during surgery is associated with an excessively superficial anaesthetic plan [68,69,70]. During surgery we observed a statistically significant higher mean heart rate in the group receiving only detomidine throughout almost the entire procedure (at T12, T14, T26, 28; TT30, TT32, T38; *p* = 0.011, 0.035, 0.049, 0.04, 0.014, 0.015, 0.022). In particular this value was statistically significantly higher from 12 min after the start of TIVA. The delayed onset of this difference was ascribed to the lack of any surgical stimulation before that moment, because around that time point the Backhaus forceps were applied to the skin.

In addition to this, in the detomidine group the systolic and mean arterial blood pressure were also higher, at T6 and T12 for MAP and at T12 for SAP (*p* = 0.022, *p* = 0.016; *p* = 0.04). The blood pressure monitoring was performed with an oscillometric noninvasive system to mimic the field conditions, where it is uncommon to have a continuous direct blood pressure monitoring technology that can detect more subtle differences [71,72].

Concerning the non-parametric variables, we observed lower myorelaxation at T14 in donkeys in group D as well as a more intense palpebral reflex (*p* = 0.027 and *p* = 0.033). Since we used dissociative drugs, nystagmus and palpebral reflex were always present [73], but when tested 14 min after the start of TIVA, the latter was more intense in the detomidine group.

Although we expected to observe significant differences between the groups, when we examined the moments of the surgical procedure that we considered more painful (skin incision, traction, ligation and emasculation of the spermatic cord), both groups showed similar trends regarding all variables. Data concerning the sympathetic response to surgical stimulation in horses clearly state that the fastest response to noxious stimulation involves an increase in heart rate and blood pressure [74]. It is likely that this may not be applicable to the donkeys, who may require a longer time to show significant variations of these parameters or may show as a first sign an increase of muscle rigidity. In fact, when we looked at the variables in the moments following the surgical stimulation, we could observe significant rise of either one or more parameters with a time-lapse of 90–120 s from the surgical stimulus.

The absence of any statistically significant difference in respiratory rate between groups can be ascribed to the moderate central nervous system depression, and better tissue perfusion compared to inhalation anaesthesia [28]. Since the type and rate of the infusion were the same in the experimental groups it is likely that the degree of the sympathetic tone inhibition mediated by the infusion itself was similar.

A NSAID was not administered before surgery to limit any influence of the response to the surgical treatment in both groups. For the same reason we decided not to use any loco-regional anaesthesia, which is well known to reduce intra and post-operative pain in case of orchiectomy in many species [71,75,76,77,78,79]. Nevertheless, all animals received a NSAID directly after the end of surgery and for the following 3 days.

Blood gas analysis was not available for all patients, and unfortunately it was not possible to have all the available samples retrieved at the same time after induction of anaesthesia, so data were not analyzed. TIVA with detomidine+ ketamine+ guaifenesin provided better cardiovascular and respiratory function compared to inhalant anaesthesia for castration in ponies [80]. Despite this, the lack of blood gas analysis data can be considered a limit of the study. A moderate increase in CO_2_ causes beneficial systemic effects, increasing cardiac output, tissue perfusion and epinephrine release. More severe increases on the other hand are responsible for hypertension and have a negative effect on blood pH, cardiac rhythm, and intracranial pressure [81].

In accordance with the increased muscle rigidity, heart rate, systolic and mean blood pressure, we also observed that donkeys in group D needed a statistically significantly higher number of top-up anaesthesia doses, presumably because of the greater intraoperative pain perception. Since we used dissociative drugs, the use of ketamine was limited to excessively evident nystagmus. In our study, the most frequently used top-ups were with thiopental (*p* = 0.012), which was administered when poor muscle relaxation was detected. Top-ups were preferred over the increase in the TIVA rate because their effects were faster in deepening of anaesthesia compared to the CRI rate. Moreover, high amounts of infused guaifenesin may cause prolonged recovery due to the cumulative properties of this drug [25].

Although the time to prepare the patients and the total anaesthesia time were not different between the groups (although longer for the detomidine group), we observed a statistically significant longer surgical time in the group of donkeys receiving only detomidine. This may be because in the detomidine group more interruptions were required for rescue treatments to allow deepening of anaesthesia.

In accordance with previous literature [70,82], both groups exhibited an excellent recovery. Donkeys’ recovery tends to be smoother than horses and they lie more quietly until able to stand, without the need for sedation [1]. When butorphanol was used, a longer time was required to completely recover from anaesthesia. As in previous studies in ponies, it may be that the donkeys receiving butorphanol were more comfortable in the immediate post-operative period. Alternatively, the association of butorphanol with detomidine may have had a synergistic effect on sedation compared to alpha-2 agonists alone [13,22,41,64]. This aspect of the protocol needs to be considered in field settings: although prolonged recovery gives the time for the patients to metabolize drugs and to recover with less ataxia, the availability of monitoring and safety equipment for a long time is not always ideal.

When we analyzed the variables for within-group comparison, we did not observe significant differences during the surgical procedure, suggesting that the anaesthestic plan was stable and safe, although more superficial in the detomidine group. The only exception to this was the degree of muscle relaxation (*p* = 0.03) that was observed in this group from 14 min after the start of TIVA up to the end of the procedure, suggesting a reaction to a surgical stimulus at that time followed by a progressive deepening of the anaesthetic plan, probably as an effect of the additional boluses of anaesthetic drugs.

Although the limited number of patients reduced the statistical power of the study, we could appreciate some significant differences between the experimental groups. As said before the lack of blood gas data may have led to underestimation of the cardiovascular function, even though the protocol is proven to be safer than inhalation anaesthesia [24].

## 5. Conclusions

We can conclude that the addition of butorphanol to detomidine for sedation of donkeys before general anaesthesia for surgical castration with a TIVA with guaiphenesin-ketamine-detomidine is beneficial. It provides a good analgesic effect and allows the administration of low dose of detomidine and anaesthetic drugs, with a more stable anaesthetic plan, lower number of top-ups drugs and consequently a safer anaesthesia. This is of crucial importance under field conditions where monitoring and safety equipment are often lacking.

## Figures and Tables

**Table 1:** Age, body weight, duration of anaesthesia, patient preparation, surgical time and recovery time in the two groups. Values are expressed as mean ± SD (SD: standard deviation). Statistical differences are expressed by the same letter on the same column (a: *p* = 0.016; b: *p* = 0.003).

	Age (Months)	Body Weight (kg)	Anaesthesia Duration (min)	Patients Preparation (min)	Surgical Time (min)	Recovery Time (min)
Group D	25 ± 6	158 ± 46	58 ± 16	17 ± 5	41 ± 9 ^a^	15 ± 10 ^b^
Group DB	22 ± 10	157 ± 73	50 ± 11	16 ± 7	29.5 ± 8.6 ^a^	80 ± 38 ^b^

## Data Availability

Data available on request due to privacy restriction. The data presented in this study are available on request from the corresponding author.

## References

[1] Matthews N.S., Taylor T.S., Hartsfield S.M. (1997). Anaesthesia of donkeys and mules. Equine Vet. Educ..

[2] Mendoza F.J., Perez-Ecija A., Toribio R.E. (2019). Clinical Pharmacology in Donkeys and Mules. Vet. Clin. N. Am. Equine Pract..

[3] Matthews N., van Loon J.P.A.M. (2019). Anesthesia, sedation, and pain management of donkeys and mules. Vet. Clin. N. Am Equine Pract..

[4] Fielding D., Fielding D., Pearson R.A. (1991). The number and distribution of equine in the world. Donkeys, Mules and Horses in the Tropical Agricoltural Devel-opment.

[5] Sgorbini M., Bonelli F., Rota A., Baragli P., Marchetti V., Corazza M. (2013). Hematology and clinical chemistry in Amiata don-key foals from birth to 2 months of age. J. Equine Vet. Sci..

[6] Ali A.B., Matoock M., Fouad M.A., Heleski C.R. (2015). Are mules or donkeys better adapted for Egyptian brick kiln work? (Until we can change the kilns). J. Vet. Behav..

[7] De Rose P., Cannas E., Cantiello P.R. (2011). Donkey-assisted rehabilitation program for children: A pilot study. Ann. Dell’Istituto Super. Sanità.

[8] Borioni N., Marinaro P., Celestini S., Del Sole F., Magro R., Zoppi D., Mattei F., Armi V.D., Mazzarella F., Cesario A. (2011). Effect of equestrian therapy and onotherapy in physical and psycho-social performances of adults with intellectual disability: A preliminary study of evaluation tools based on the ICF classification. Disabil. Rehabil..

[9] Lizarraga I., Sumano H., Brumbaugh G.W. (2010). Pharmacological and pharmacokinetic differences between donkeys and horses. Equine Vet. Educ..

[10] Grosenbaugh D.A., Reinemeyer C.R., Figueiredo M.D. (2011). Pharmacology and therapeutics in donkeys. Equine Vet. Educ..

[11] Matthews N.S., Peck K.E., Mealey K.L., Taylor T.S., Ray A.C. (1997). Pharmacokinetics and cardiopulmonary effects of guaifenesin in donkeys. J. Vet. Pharmacol. Ter..

[12] Jordan W.J., Svendsen E.D. (1986). Surgery. The professional Handbook of the Donkeys.

[13] Joubert K., Briggs P., Gerber D., Gottschalk R. (1999). The sedative and analgesic effects of detomidine-butorphanol and detomidine alone in donkeys. J. S. Afr. Vet. Assoc..

[14] Daunt D.A. (1994). Detomidine in equine sedation and analgesia. Compend. Contin. Educ..

[15] Yamashita K., Tsubakishita S., Futaoka S., Ueda I., Hamaguchi H., Seno T., Katoh S., Izumiawa Y., Kotani T., Miur W.W. (2000). Cardiovascular effect of medetomidine, detomidine and xylazine in horses. J. Vet. Med. Sci..

[16] Mostafa M.B., Farag K.A., Zomor E., Bashandy M.M. (1995). The Sedative and Analgesic Effects of Detomidine (Domosedan) in Donkeys. J. Vet. Med. Ser. A.

[17] El-Maghraby H.M., Atta A.H. (1997). Sedative and analgesic effects of detomidine with and without butorphanol in donkey. Assiut. Vet. Med. J..

[18] Lavoie J.P., Phan S.T., Blais D. (1996). Effects of a combination of detomidine and butorphanol on respiratory function in horses with or without chronic obstructive pulmonary disease. Am. J. Vet. Res..

[19] Robertson J.T., Muir W.W. (1983). A new analgesic drug combination in the horse. Am. J. Vet. Res..

[20] Taylor P.M., Browning A.P., Harris C.P. (1988). Detomidine-butorphanol sedation in equine clinical practice. Vet. Rec..

[21] Clarke K.W., Taylor P.M. (1986). Detomidine: A new sedative for horses. Equine Vet. J..

[22] Clarke K.W., Paton B.S. (1988). Combined use of detomidine with opiates in the horse. Equine Vet. J..

[23] England G.W., Clarke K.W. (1996). Apha2 adrenoreceptor agonists in the horse—A review. Br. Vet. J..

[24] Kalpravidh M., Lumb W.V., Wright M., Heath R.B. (1984). Effects of butorphanol, flunixin, levorphanol, morphine and xylazine in ponies. Am. J. Vet. Res..

[25] Coelho C.M.M., Moreno J.C.D., da Goulart D.S., Caetano L.B., Soares L.K., Coutinho G.H., Alves G.E.S., da Silva A.F. (2014). Evaluation of cardiorespiratory and biochemical effects of ketamin–propofol and guaiphenesin-ketamine-xylazine anaesthesia in donkeys (*Equus asinus*). Vet. Anaesth. Analg..

[26] Taylor P.M., Luna S.P.L., Sear J.W., Wheeler M.J. (1996). Total intravenous anaesthesia in ponies using detomidine ketamine ad guaiphenesin: Pharmacokinetics, cardiopulmonary and endocrine effects. Res. Vet. Sci..

[27] Bettschart-Wolfensberger R., Taylor P.M., Sear J.W., Bloomfield M.R., Rentsch K., Dawlin S. (1996). Physiological effects of anaesthesia induced and maintained by intravenous administration of a climazolam-ketamine combination in ponies premedicated with acepromazine and xylazine. Am. J. Vet. Res..

[28] Taylor P.M., Kirby J.J., Shrimpton D.J., Johnson J.B. (1998). Cardiovascular effects of surgical castration during anaesthesia main-tained with halothane or infusion of detomidine, ketamine and guaifenesin in ponies. Equine Vet. J..

[29] Muir W.W., Lerche P., Robertson J.T., Hubbel J.A., Beard W., Miller T., Badgley B., Bothwell V. (2000). Comparison of four drugs combinations for total intravenous anaesthesia of horses undergoing surgical removal of abdominal testis. J. Am. Vet. Med. Ass..

[30] Matthews N.S., Taylor T.S., A Sullivan J. (2002). A comparison of three combinations of injectable anesthetics in miniature donkeys. Vet. Anaesth. Analg..

[31] Emami M.R., Seifi H., Tavakoli Z. (2006). Effects of totally intravenous thiopental anesthesia on cardiopulmonary and termoregu-latory system in donkeys. J. Appl. An. Res..

[32] Naddaf H., Baiadam A., Rasekh A., Arasteh A., Saboza S. (2015). Cardiopulmonary effects during anaesthesia induced and mantained with propofol in acepromazine premedicated donkey. Vet. Anaesth. Analg..

[33] Amin A.A., Mohammed M.S. (2012). Cardiopulmonary effects of detomidine-propofol and ketamine administration in the donkey. Al Anbar J. Vet. Sci..

[34] Lindsay F.E., Clayton H.M. (1986). An anatomical and endoscopic study of the nasopharynx and larynx of the donkeys (*Equus asinus*). J. Anat..

[35] Sprayson T., Thiemann A. (2007). Clinical approach to castration in the donkey. Practice.

[36] Lerche P. (2013). Total intravenous anaesthesia in horses. Vet. Clin. N. Am. Equine Pract..

[37] Lizarraga I., Castillo-Alcala F., Varner K.M., Robinson L.S. (2015). Sedation and mechanical antinociception after intravenous administration of detomidine in donkeys: A dosage-effect study. Vet. Rec..

[38] Hamed M.A., Abouelnasr K.S., Ibrahim H.M., El-Khodery S.A. (2017). Comparative, Sedative, and Analgesic Effects of Epidural Dexmedetomidine and Xylazine in Donkeys (*Equus asinus*). J. Equine Vet. Sci..

[39] Kerr C.L., McDonnell W.N., Young S.S. (1996). A comparison of romifidine and xylazine when used with diazepam/ketamine for short duration anaesthesia in the horse. Can. Vet. J..

[40] Vesce G. (1982). Ocular nystagmus during general anaesthesia in the horse. Vet. Anaesth. Analg..

[41] Love E.J., Taylor P.M., Clark C., Whay H.R., Murrell J. (2009). Analgesic effect of butorphanol in ponies following castration. Equine Vet. J..

[42] Love E.J., Taylor P.M., Whay H.R., Murrell J. (2013). Postcastration analgesia in ponies using buprenorphine hydrochloride. Vet. Rec..

[43] Costa E.D., Minero M., Lebelt D., Stucke D., Canali E., Leach M.C. (2014). Development of the Horse Grimace Scale (HGS) as a Pain Assessment Tool in Horses Undergoing Routine Castration. PLoS ONE.

[44] Mellor D., Stafford K. (2000). Acute castration and/or tailing distress and its alleviation in lambs. N. Z. Vet. J..

[45] Aengwanich W., Sakundech K., Chompoosan C., Tuchpramuk P., Boonsorn T. (2019). Physiological changes, pain stress, oxidative stress, and total antioxidant capacity before, during, and after castration in male dogs. J. Vet. Behav..

[46] Mogheiseh A., Koohi F., Nazifi S., Tabrizi A.S., Taheri P., Salavati S. (2019). Oxidative-antioxidative status and hepatic and renal factors following melatonin administration in castrated and intact dogs. Basic Clin. Androl..

[47] Abou-Khalil N.S., Ali M.F., Ali M.M., Ibrahim A. (2020). Surgical castration versus chemical castration in donkeys: Response of stress, lipid profile and redox potential biomarkers. BMC Vet. Res..

[48] Taylor P.M. (1985). Chemical restraint of the standing horse. Equine Vet. J..

[49] Whiteheads G., French J., Ikin P. (1991). Welfare and veterinary care of donkeys. Practice.

[50] Lizarraga I., Castillo-Alcada F., Varner K.M., Robinson L.S. (2016). Sedation and mechanical hypoalgesia after sublingual admin-istration of detomidine hydrochloride gel to donkeys. J. Am. Vet. Med Assoc..

[51] Samimi A.S. (2019). Evaluation of the sedative and clinical effects of xylazine, detomidine, medetomidine and dexmedetomidine in miniature donkeys. N. Z. Vet. J..

[52] El-Kammar M.H., Gad S.B. (2014). Antagonism of detomidine-induced sedation, analgesia, clinicophysiological, and biochemical effects in donkeys using IV tolazoline or atipamezole. J. Equine Vet. Sci..

[53] Schatzmann U., Armbruster S., Stucki F., Busato A., Kohler I. (2001). Analgesic Effect of Butorphanol and Levomethadone in Detomidine Sedated Horses. J. Vet. Med. Ser. A.

[54] Kruluc P., Nemec A. (2006). Electroencephalographic and electromyographic changes during the use of detomidine and detomidine-butorphanol combination in standing horses. Acta Vet. Hung..

[55] Nyman G., Marntell S., Edner A., Funkquist P., Morgan K., Hedenstierna G. (2009). Effect of sedation with detomidine and butorphanol on pulmonary gas exchange in the horse. Acta Vet. Scand..

[56] Knych H.K., Casbeer H.C., McKemie D.S., Arthur R.M. (2013). Pharmacokinetics and pharmacodynamics of butorphanol following intravenous administration to the horse. J. Vet. Pharmacol. Ther..

[57] Marly C., Bettschart R., Nussbaumer P., Moine S., Ringer S.K. (2014). Evaluation of a romifidine constant rate infusion protocol with or without butorphanol for dentistry and ophthalmologic procedures in standing horses. Vet. Anaesth. Analg..

[58] Chiavaccini L., Claude A.K., Lee J.H., Ross M.K., Meyer R.E., Langston V.C. (2015). Pharmacokinetics and pharmacodynamics comparison between subcutaneous and intravenous butorphanol administration in horses. J. Vet. Pharmacol. Ther..

[59] Moorman V.J., Bass L., King M.R. (2019). Evaluation of the effects of commonly used α2-adrenergic receptor agonists alone and in combination with butorphanol tartrate on objective measurements of lameness in horses. Am. J. Vet. Res..

[60] De Grauw J., van Loon T. (2020). Clinical effects of two doses of butorphanol with detomidine for intravenous premedication of healthy warmblood horses. Vet. Anaesth. Analg..

[61] Paine S.W., Bright J., Scarth J.P., Hincks P.R., Pearce C.M., Hannan C., Machnik M., Hillyer L. (2020). The intravenous pharmacokinetics of butorphanol and detomidine dosed in combination compared with individual dose administrations to exercised horses. J. Vet. Pharmacol. Ther..

[62] Muir W.W., Robertson J.T. (1985). Visceral analgesia: Effects of xylazine, butorphanol, meperidine, and pentazocine in horses. Am. J. Vet. Res..

[63] Bidwell L.A. (2010). How to anesthetize donkeys for surgical procedures in the field. AAEP Proc..

[64] Lizarraga I., Castillo-Alcala F. (2015). Sedative and mechanical hypoalgesic effects of butorphanol in xylazine -premedicated don-keys. Equine Vet. J..

[65] El-Kammar M.H., Gad S.B. (2014). Evaluation of the sedative, analgesic, clinicophysiological and haematological effects of intra-venous detomidine, detomidine-butorphanol, romifidine and romifidine-butorphanol in standing donkeys. Eq. Vet. Educ..

[66] Van Dierendonck M.C., Burden F.A., Rickards K., Van Loon J.P. (2020). Monitoring Acute Pain in Donkeys with the Equine Utrecht University Scale for Donkeys Composite Pain Assessment (EQUUS-DONKEY-COMPASS) and the Equine Utrecht University Scale for Donkey Facial Assessment of Pain (EQUUS-DONKEY-FAP). Animals.

[67] Van Loon J.P., de Grauw J.C., Burden F., Vos K.J., Bardelmeijer L.H., Rickards K. (2021). Objective assessment of chronic pain in donkeys using the donkey chronic pain scale (DCPS): A scale-construction study. Vet. J..

[68] Weary D., Niel L., Flower F.C., Fraser D. (2006). Identifying and preventing pain in animals. Appl. Anim. Behav. Sci..

[69] Haga H.A., Dolvik N.I. (2005). Electroencephalographic and cardiovascular variables as nociceptive indicators in isoflurane-anaesthetized horses. Vet. Anaesth. Analg..

[70] Suriano R., Varasano V., Robbe D., Carluccio A., Straticò P., Contri A., Petrizzi L. (2014). Intraoperative analgesic effect of intrafunicolar lidocaine injection during orchiectomy in isoflurane anaesthetized Martina Franca donkeys. J. Eq. Vet. Sci..

[71] Hubbel J.A.E., Muir W.W. (2009). Monitoring anaesthesia. Equine Anaesthesia, Monitoring and Emergency Therapy.

[72] Yamaoka T., Flaherty D., Pawson P., Scott M., Auckburally A. (2017). Comparison of arterial blood pressure measurements ob-tained invasively or oscillometrically using a Datex S/5 compact monitor in anaesthetized adult horses. Vet. Anaesth. Analg..

[73] El-Ghoul W., Zabady M., Saleh I. (2004). Total intravenous anaesthesia in donkeys (equus asinus): Comparison of anaesthetic and cardiorespiratory effects of four anaesthetic drug combinations. Vet. Med. J. Giza.

[74] Haga H.A., Ljkkjen S., Revold T., Ranheim B. (2006). Effect of intratesticular injection of lidocaine on cardiovascular responses to castration in isoflurane-anaesthetized stallions. Am. J. Vet. Res..

[75] Wood G.N., Molony V., Fleetwood-Walker S.M., Hodgson J.C., Mellor D.J. (1991). Effects of local anesthesia and intravenous naloxone on the changes in behaviour and plasma concentration of cortisol produced by castration and tail docking with tight rubber rings in young lambs. Res. Vet. Sci..

[76] Stafford K.J., Mellor D.J., Todd S.E., Bruce R.A., Ward R.N. (2002). Effects of local anaesthesia or local anaesthesia plus a non-steroidal an-ti-inflammatory drug on the acute cortisol response of calves to five different methods of castration. Res. Vet. Sci..

[77] A Haga H., Ranheim B. (2005). Castration of piglets: The analgesic effects of intratesticular and intrafunicular lidocaine injection. Veter. Anaesth. Analg..

[78] Straticò P., Varasano V., Suriano R., Mariscoli M., Robbe D., Giammarco M., Vignola G., Petrizzi L. (2018). Analgesic effects of intravenous flunixin and intrafunicular lidocaine or their combination for castration of lambs. Veter. Rec. Open.

[79] Portier K.G., Jaillardon L., Leece E.A., Walsh C.M. (2009). Castration of horses under total intravenous anaesthesia: Analgesic effects of lidocaine. Vet. Anaesth. Analg..

[80] McMurphy R.M., Young L.E., Marlin D.J., Walsh K. (2002). Comparison of the cardiopulmonary effects of anaesthesia in horses. J. Vet. Res..

[81] E Wagner A., Bednarski R.M., Muir W.W. (1990). Hemodynamic effects of carbon dioxide during intermittent positive-pressure ventilation in horses. Am. J. Vet. Res..

[82] Matthews N., Van Loon J.P.A.M. (2011). Anaesthesia and analgesia of the donkey and the mule. Equine Vet. Educ..

